# Synthesis and Characterization of Monodispersed Spherical Calcium Oxide and Calcium Carbonate Nanoparticles via Simple Pyrolysis

**DOI:** 10.3390/nano12142424

**Published:** 2022-07-15

**Authors:** Raji Atchudan, Suguna Perumal, Jin Joo, Yong Rok Lee

**Affiliations:** 1 Department of Applied Chemistry, Kyungpook National University, Daegu 41566, Korea; 2 School of Chemical Engineering, Yeungnam University, Gyeongsan 38541, Korea; 3 Department of Chemistry, Saveetha School of Engineering, Saveetha Institute of Medical and Technical Sciences, Chennai 602105, Tamil Nadu, India; 4 Department of Chemistry, Sejong University, Seoul 143747, Korea; suguna.perumal@gmail.com

**Keywords:** calcium carbonate, calcium oxide, calcium hydroxide, calcium oleate, carbonization, pyrolysis

## Abstract

In this study, calcium carbonate nanoparticles (CCNPs) and calcium oxide nanoparticles (CONPs) are synthesized by the carbonization/calcination of calcium oleate. CONPs are an essential inorganic material, and they are used as catalysts and as effective chemisorbents for toxic gases. CCNPs are widely used in plastics, printing ink, and medicines. Here, calcium oleate is used as a starting material for the preparation of CCNPs and CONPs. This calcium oleate is prepared from calcium hydroxide and oleic acid in ethanol under mild reflux conditions. The effect of the calcination temperature of calcium oleate is examined during the synthesis of CCNPs and CONPs. By simple carbonization/calcination, calcite-type CCNPs and CONPs are prepared at <550 °C and >600 °C, respectively. The synthesized nanomaterials are analyzed by various physicochemical characterization techniques such as X-ray diffraction (XRD), Fourier transform infrared spectroscopy, thermogravimetric analysis (TGA) with derivative thermogravimetry (DTG), and scanning electron microscopy (SEM) with energy dispersive X-ray analysis. An X-ray diffractometer and the Scherrer formula are used to analyze the crystalline phase and crystallite size of prepared nanoparticles. TGA techniques confirm the thermal stability of the calcium oleate, CCNPs, and CONPs. The SEM analysis illustrates the dispersive behavior and cubic/spherical morphologies of CCNPs/CONPs. Furthermore, the obtained results are compared to the CCNP and CONP samples prepared using calcium hydroxide. As a result, the carbonization/calcination of calcium oleate produces monodispersed CONPs, which are then compared to the CONPs from calcium hydroxide. Additionally, from calcium oleate, CONPs can be prepared on a large scale in a cheap, convenient way, using simple equipment which can be applied in various applications.

## 1. Introduction

Calcium carbonate (CaCO_3_) is one of the most important inorganic materials because it has a wide range of applications in various fields of industry [1,2,3]. Especially, it is used in white pigments, fillers, biomedical implanting, drug delivery, and bone regeneration applications. Furthermore, CaCO_3_ is used in the preparation of paper, rubber, paint, and plastics [4,5]. Three polymorphic forms of CaCO_3_ exist, and they are listed as follows in order of decreasing thermodynamic stability: rhombohedral calcite, orthorhombic aragonite, and hexagonal vaterite [6]. Most commonly, CaCO_3_ nanoparticles (CCNPs) are synthesized through the wet chemical precipitation technique. However, the technique causes the considerable agglomeration of particles during synthesis, leading to a bimodal size distribution [7,8,9]. Moreover, the technique requires the precise control of a number of operation parameters, such as reagent concentration, residential time, stirring speed, pH, feed point position, and temperature, which determine the size, crystal structure, and morphology of the particles. Apart from the wet chemical precipitation method, CCNPs can be prepared by several other methods, including emulsion liquid membrane, gas–liquid carbonation, and the gas–liquid microdispersion process [10]. The emulsion liquid membrane has some limitations, such as emulsion instability, the use of surfactants, and the need to recover the transported species by breaking the emulsion. Notably, the gas–liquid microdispersion process also has some drawbacks, including limited mass transfer. Among these methods, the gas–liquid carbonation method is one of the best industrial processes for the preparation of CCNPs.

Calcium oxide (CaO) is applied in various fields because it is inexpensive, non-corrosive, and easy to handle with excellent basicity compared to typical homogeneous base catalysts, such as catalysts, bactericides, additives in refractories, flue gas desulfurization, pollutant emission control, and particularly critical adsorbents for toxic chemical agents [11,12]. Apart from these, CaO has been identified as the most promising candidate for CO_2_ capture because CaO-based adsorbents have the following advantages: a high reactive sorption capacity for CO_2_, a low cost, and an abundance of natural precursors [13,14,15]. CaO is used as a starting material for the preparation of CaCO_3_ in reversible reactions (Equation (1)). The calcination process determines the structural characteristics of CaO, which is an active sorbent for CO_2_ [13]. CaO nanoparticles (CONPs) are generally prepared by several methods, including thermal decomposition, microwave processes, sonochemical synthesis, hydrogen plasma–metal reactions, solvothermal reactions, precipitation, and water-in-oil microemulsions [12]. The above methods have some limitations, such as the use of toxic organic solvents, prolonged reaction time, high synthesis temperature, and expensive/intricate equipment. In our previous study, monodisperse CONPs were obtained from a calcium oleate precursor through the thermal decomposition of calcium oleate. Typically, calcium stearate and oleic acid are placed in a reaction vessel and heated at 100 °C for 1 h under vacuum to remove the oxygen and moisture from the mixture. Then, the temperature is increased to 350 °C and maintained for 5 h with vigorous stirring under an argon atmosphere. After cooling, an excess amount of ethanol is added to the final reaction mixture, yielding a brownish residue. The product of calcium oleate (brownish residue) is recovered by centrifugation and washing with ethanol several times to remove the excess amount of unreacted oleic acid. This calcium oleate precursor is prepared by multistep synthesis through a laborious process, and prohibitive reagents are used [16].

(1) $$\mathrm{CaO}\left(s\right)+{\;\mathrm{CO}}_{2}\left(g\right)\begin{array}{c} \overset{\mathrm{Carbonization}}{\to }\\ \underset{\mathrm{Calcination}}{\leftarrow } \end{array}{\;\mathrm{CaCO}}_{3}\left(s\right)$$

Here, a new simple way is proposed to prepare CCNPs and CONPs from calcium oleate. Calcium oleate is prepared from calcium hydroxide and oleic acid in the presence of ethanol under mild reflux conditions. The effect of reaction temperature on the formation of CCNPs and CONPs is investigated systematically. The structural properties of CCNPs and CONPs are examined by X-ray diffraction (XRD), Fourier transform infrared (FTIR) spectroscopy, thermogravimetric analysis (TGA) with derivative thermogravimetry (DTG), and scanning electron microscopy (SEM) with energy dispersive X-ray analysis (EDAX).

## 2. Experimental Conditions

### 2.1. Required Materials

Calcium hydroxide (99.9%), ethanol (99.8%), and oleic acid (90%) were purchased from Aldrich Chemicals, and they were used as received without any further purification.

### 2.2. Preparation of Calcium Oleate, CCNPs, and CONPs

**Scheme 1:**
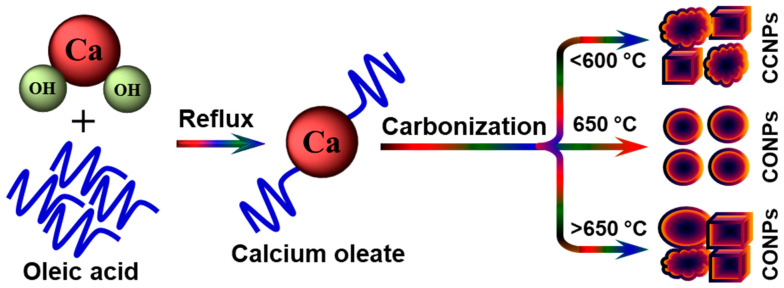
Formation of calcium oleate and CCNPs/CONPs by simple solvothermal and carbonization/calcination methods.

An appropriate amount of calcium hydroxide (1 mol) and oleic acid (2.5 mol) was added to ethanol. The reaction mixture was refluxed for 5 h, and the obtained solid material (calcium oleate) was filtered and washed several times with ethanol. Subsequently, the calcium oleate was dried and then carbonized/calcined at various temperatures for the production of CCNPs and CONPs. In a typical growth experiment, an appropriate amount of calcium oleate was taken into a silica crucible, and it was carbonized/calcined for 5 h in a muffle furnace at different temperatures (450–800 °C) under static air conditions. The muffle furnace was cooled to room temperature, and then the final product (white color powder) was collected from the crucible. **Scheme 1** clearly illustrates the formation of calcium oleate by a mild reflux condition and the preparation of CCNPs/CONPs from calcium oleate via the simple carbonization/calcination (pyrolysis) method. On the other hand, CCNPs/CONPs were prepared using calcium hydroxide. For this, an appropriate amount of calcium hydroxide was taken alone into a silica crucible, and it was calcined for 5 h in a muffle furnace at different temperatures (450–800 °C) under static air conditions. The formation of CCNPs and CONPs from calcium hydroxide via the simple calcination method is shown in **Scheme S1**.

## 3. Results and Discussion

**Figure 1:**
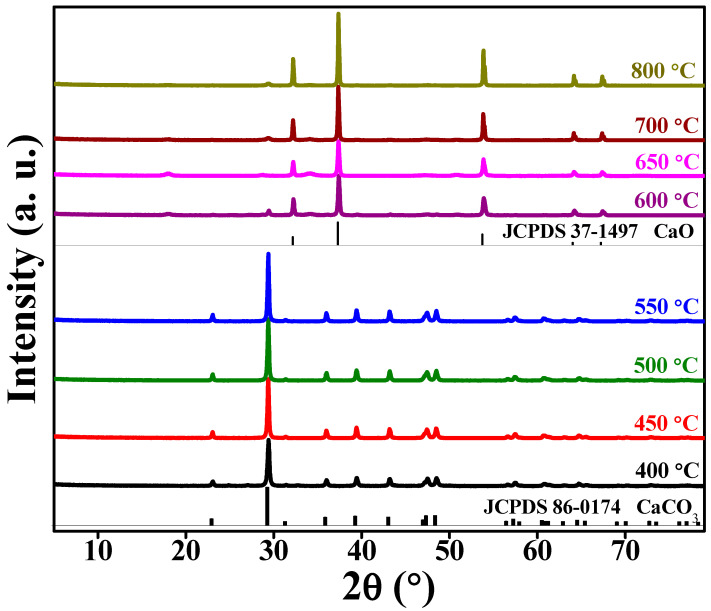
XRD patterns of CCNPs and CONPs fabricated from calcium oleate at different carbonization/calcination temperatures.

The XRD patterns of CCNPs and CONPs are shown in **Figure 1**. The patterns exhibited an intense diffraction peak around 29.4° (2θ) due to (104) diffraction and low intense peaks around 23.1, 31.4, 36.0, 39.4, 43.2, 47.1, 47.4, 48.5, 56.6, 57.5, 60.7 and 64.8° (2θ) attributed to the (012), (006), (110), (113), (202), (024), (018), (116), (211), (112), (122) and (220) planes, respectively, confirming the formation of CCNPs with the calcite polymorph phase [10,17]. The obtained materials possessed a well-defined crystalline structure and were found to be in good agreement with a standard JCPDS pattern (JCPDS card no: 86-0174) for similar calcite nanoparticles [18]. The XRD patterns of the CCNPs prepared from calcium oleate between the carbonization temperatures of 400 and 550 °C had similar phase compositions. However, with increasing carbonization temperature, the peak intensities also increased, which might be due to an increase in the degree of crystallinity with temperature. The crystallite size of the calcite, which is estimated by the Debye–Scherrer equation from the unique peak (104) plane, was around 100 nm for the samples obtained at 550 °C. The CCNP phases were turned into CONPs phases beyond 600 °C and were confirmed by the XRD patterns with peaks at 32.2, 37.4, 53.9, 64.2, and 67.4° (2θ) corresponding to the (111), (200), (202), (311) and (222) planes, respectively. These patterns agreed well with the corresponding standard values given in the JCPDS pattern of CaO (JCPDS card no: 37-1497) [19,20]. The intensities of the diffraction patterns increased with increasing calcination temperature from 600 to 800 °C without any further phase changes. The Debye–Scherrer equation was used to estimate the crystallite size using the faces of the (200) peaks. The sizes were calculated as about 65 and 75 nm for the samples obtained at 600 and 650 °C, respectively.

**Figure 2:**
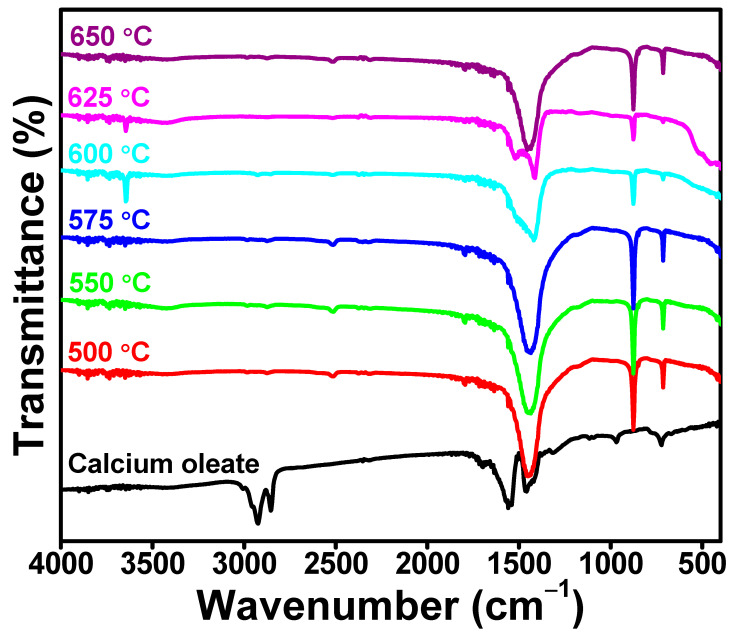
FTIR spectra of synthesized calcium oleate; CCNPs and CONPs from calcium oleate at different carbonization/calcination temperatures.

FTIR spectra of synthesized calcium oleate, CCNPs, and CONPs were recorded to determine their existing surface functionalities. The infrared spectra of the calcium oleate, CCNPs, and CONPs are shown in **Figure 2**. As seen in the spectra of the CCNPs, three strong bands centered at around 713, 875, and 1450 cm^−1^, corresponding to framework vibrations of ν_4_, ν_3_, and ν_2_, respectively, and were considered to arise from CO_3_^2−^ [21]. Among these vibrations (ν_4_, ν_3,_ and ν_2_), ν_2_ vibration was strong and broad due to the crystalline structure of CONPs and CCNPs without major defects [22]. The weak and broad peaks around 3440 cm^−1^ may be attributed to the stretching vibration of −OH groups of physically adsorbed or lattice water molecules [23,24,25]. Deformational vibrations of the adsorbed molecules caused absorption bands around 1635 cm^–1^. The intensities of the ν_4_, ν_3,_ and ν_2_ vibrations decreased for CONPs prepared at 600 and 625 °C, which might be due to the transformation of CCNPs into CONPs. In addition, a sharp band was observed at 3645 cm^–1^ and attributed to the asymmetric –OH stretching vibration [16]. This asymmetric –OH stretching vibration decreased with increasing reaction temperature from 600 to 625 °C and then disappeared at 650 °C, indicating the degree of the modification. The FTIR spectra of calcium oleate showed strong peaks at 2924 and 2854 cm^−1^, corresponding to C−H asymmetric and symmetric vibrations in the methyl groups, respectively, which were not observed in the CCNPs and CONPs. The small peak at 1705 cm^−1^ is assigned to the −C=O stretching vibration of carboxylate groups in the calcium oleate [26]. The intense bands between 1510 and 1610 cm^−1^ were due to asymmetric stretching bands for uni- and bidentate carboxylate groups associated with calcium ions [27]. The hydrogen bonding of fatty acids was damaged during the calcination of calcium oleate at a suitable temperature, resulting in the formation of CCNPs and CONPs [28].

**Figure 3:**
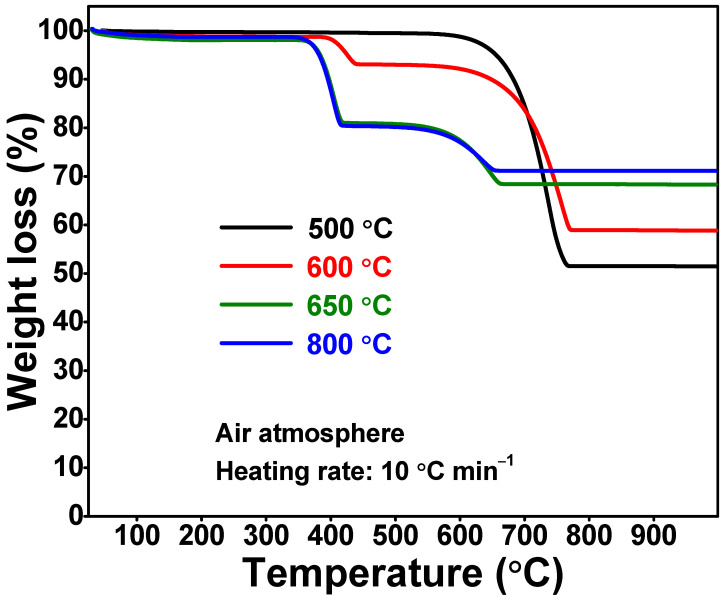
TGA curves of CCNPs and CONPs from calcium oleate at different carbonization/calcination temperatures.

The thermal stability of synthesized calcium oleate was examined by TGA, and the corresponding results are shown in **Figure S1**. The TGA curves of calcium oleate displayed four distinct weight losses. The initial weight loss was up to 150 °C due to the removal of physically adsorbed water molecules over the surface. The second weight loss was observed between 150 and 350 °C, and was associated with removing functional groups of calcium oleate. Third, weight loss at 350–520 °C was due to the decomposition of oleate from calcium oleate and the formation of CCNPs, and the horizontal portion (500–570 °C) gives the range of the thermal stability of CCNPs. The fourth and final weight loss was displayed between 570 and 650 °C, indicating the decomposition of CCNPs into CONPs and CO_2_. After that, no weight loss was observed up to 1000 °C, suggesting the stability of the CONPs. This result matches well with the XRD result. The thermal decomposition of the synthesized CCNPs and CONPs was evaluated at temperatures ranging from 25 to 1000 °C using TG/DTG analysis (**Figure 3**). An initial minute quantity of weight loss below 150 °C was due to the desorption of physically adsorbed water molecules. The second/final weight loss due to the decomposition of the occluded organic template occurred between 600 and 770 °C. CCNPs were decomposed and transferred into CONPs and CO_2_ (g). The loss of around 47% of the initial mass in this temperature range was due to the escape of CO_2_ from the system after the decomposition of CCNPs. Furthermore, we observed the absence of decomposition up to 1000 °C from the thermogram, which is credited to the purity of the synthesized calcite nanoparticles. Two separate weight losses were displayed in the CONPs prepared at above 600 °C. The first weight loss was observed from 380 to 450 °C, and the second/final weight loss was observed from 550 to 770 °C for the CONPs prepared at 600 °C. The CONPs prepared above 650 °C showed the weight losses from 350 to 420 °C and 500 to 670 °C. The first weight loss was due to the removal of hydroxyl groups on the surface of CONPs, and the final weight loss was due to the incomplete conversion of CCNPs into CONPs during calcination at a temperature beyond 600 °C [14]. The derivative thermogravimetry (DTG) curves clearly showed the appropriate weight loss of the CCNPs and CONPs, and the corresponding DTG curves are presented in **Figure S2**.

**Figure 4:**
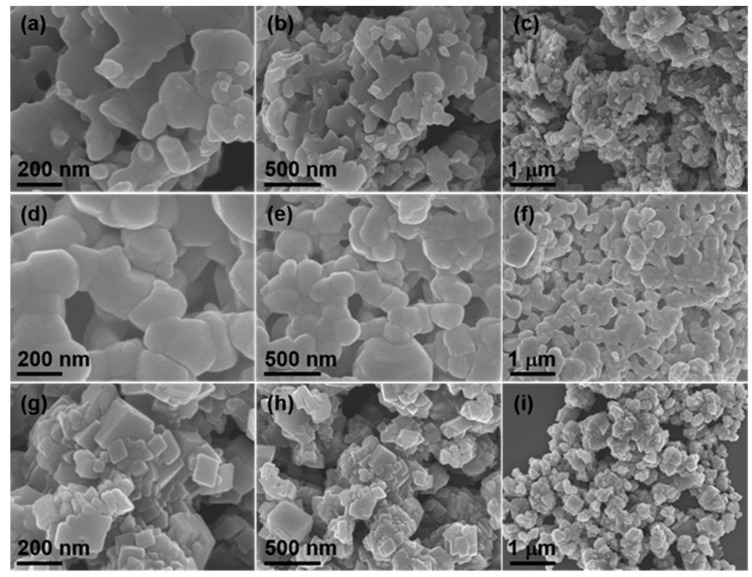
SEM images of CCNPs from calcium oleate at different temperatures: (**a**–**c**) 450 °C, (**d**–**f**) 500 °C, and (**g**–**i**) 550 °C.

**Figure 5:**
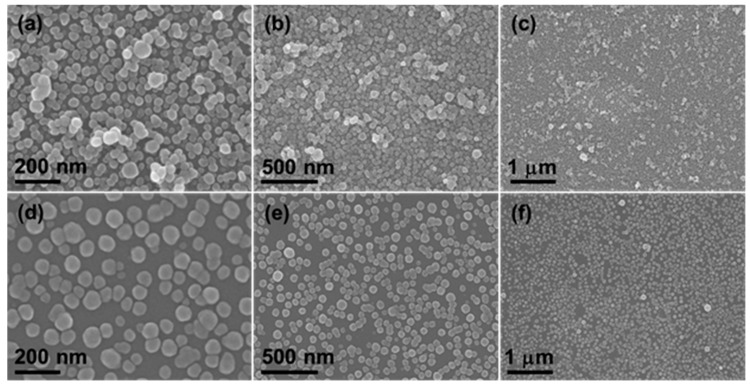
SEM images of CONPs from calcium oleate at different temperatures: (**a**–**c**) 600 °C and (**d**–**f**) 650 °C.

**Figure 6:**
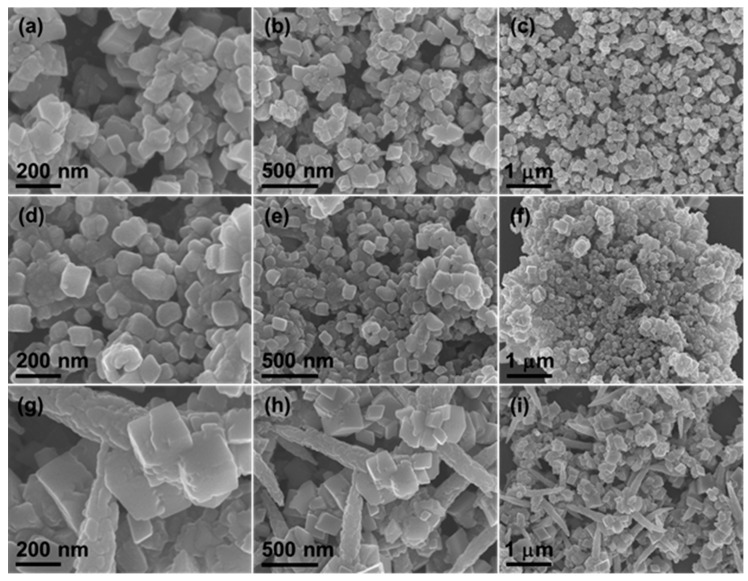
SEM images of CONPs from calcium oleate at different temperatures: (**a**–**c**) 700 °C, (**d**–**f**) 750 °C, and (**g**–**i**) 800 °C.

The morphologies of the nanoparticles were revealed by SEM analysis. **Figure 4**, **Figure 5** and **Figure 6** show the SEM images of CCNPs and CONPs synthesized at different temperatures. The experiments were carried out to study the effect of reaction temperature on the morphology of CCNPs and CONPs fabricated from calcium oleate via a simple carbonization/calcination method. The nanoparticles obtained at 600 and 650 °C were nearly spherical and uniform in size, but particles formed at 450–550 and 650–800 °C were cubic-shaped and aggregated. The nanoparticles formed at 650 °C had unique properties such as good dispersion and narrow size distribution. The diameters of the nanoparticles were in the range of about 60–80 nm. The average diameter of the CONPs was estimated as 70 nm. This result is consistent with the size calculated by the Debye–Scherrer equation in XRD studies, which implies a single crystalline nature of prepared nanoparticles. The EDAX spectra of CCNPs and CONPs were recorded for the samples synthesized from calcium oleate at different carbonization/calcination temperatures (550, 650, and 750 °C). The corresponding spectra displayed the appearance of carbon (C), oxygen (O), and calcium (Ca) elements (**Figure S3**) in the prepared CCNPs and CONPs. Carbonization/calcination temperature is one of the most significant factors for the preparation of CCNPs and CONPs.

On the other hand, CCNPs and CONPs were synthesized directly using calcium hydroxide at different carbonization/calcination temperatures (550, 650, and 750 °C), and the corresponding SEM images are shown in **Figure S4**. The transformation of calcium hydroxide into CCNPs and CONPs with respect to carbonization/calcination temperature followed the same trend as that of CCNPs and CONPs from calcium oleate. However, there was a significant difference in terms of homogeneity and the size distribution of the particles. The average diameter of the CONPs (650 °C) was measured to be at about 150 nm. This reveals that encapsulation of oleic acid in the calcium (calcium oleate) controlled the particle size and monodispersity of the CONPs. Hence, the CONPs were monodispersed when prepared from calcium oleate compared to CONPs from calcium hydroxide.

## 4. Conclusions

CCNPs and CONPs were synthesized by the simple carbonization/calcination of calcium oleate and prepared by the interaction of calcium hydroxide and oleic acid. Below 600 °C, aggregated CCNPs with calcite polymorph phase were obtained. However, sphere-shaped CONPs were obtained by the calcination at 600–650 °C, and the CONPs were well dispersed with narrow size distribution. The diameters of CONPs were virtually the same, and the range in diameter was calculated as 60–80 nm. The spherical shape of CONPs was turned into different morphologies beyond 650 °C, with moderate aggregation. Additionally, the CONPs’ particle size increased in the temperature range from 700 to 800 °C. Hence, the monodisperse CONPs were obtained from calcium oleate at optimal calcination temperature (650 °C). On the other hand, CCNPs and CONPs, as prepared from calcium hydroxide, showed polydispersed and aggregated particles. Thus, this study provides the carbonization/calcination route of calcium oleate, a versatile and simple technique that can be used for the large-scale production of CCNPs and monodispersed CONPs that can be applied in various vital and industrial applications, including papers, paints, plastics, and biomedical products.

## Data Availability

Not applicable.

## Supplementary Materials

The following supporting information can be downloaded at: https://www.mdpi.com/article/10.3390/nano12142424/s1. Scheme S1: Illustration of the formation of CCNPs and CONPs from calcium hydroxide via simple carbonization/calcination. Figure S1: TGA curve of calcium oleate was synthesized using calcium hydroxide and oleic acid. Figure S2: DTG curves of CCNPs and CONPs obtained from calcium oleate. Figure S3: EDAX spectra of (a) CCNPs (550 °C), (b) CONPs (650 °C) and (c) CONPs (750 °C) synthesized using calcium oleate. Figure S4: (a–c) SEM images of CCNPs were synthesized at 550 °C using calcium hydroxide. SEM images of CONPs synthesized at 650 °C (d–f), and 750 °C (g–i) using calcium hydroxide.
