# Supplementary material for: Synthesis and Characterization of Monodispersed Spherical Calcium Oxide and Calcium Carbonate Nanoparticles via Simple Pyrolysis
Source: Nanomaterials (Basel). 2022 Jul 15;12(14):2424. doi: 10.3390/nano12142424 (PMC9321580; doi:10.3390/nano12142424)
Supplement: Supplementary file 1 [file nanomaterials-12-02424-s001.zip › nanomaterials-1794129-supplementary.pdf]

nanomaterials

Supplementary Materials

Synthesis and Characterization of Monodispersed Spherical Calcium Oxide and Calcium Carbonate Nanoparticles Via Simple Pyrolysis

Raji Atchudan [1,2,3,*,†], Suguna Perumal [4,†], Jin Joo [1,*], Yong Rok Lee [2,*]

[1] Department of Applied Chemistry, Kyungpook National University, Daegu 41566, Republic of Korea
[2] School of Chemical Engineering, Yeungnam University, Gyeongsan 38541, Republic of Korea
[3] Department of Chemistry, Saveetha School of Engineering, Saveetha Institute of Medical and Technical Sciences, Chennai 602105, Tamil Nadu, India
[4] Department of Chemistry, Sejong University, Seoul 143747, Republic of Korea; suguna.perumal@gmail.com
* Correspondence: atchudanr@yu.ac.kr (R.A.); joojin@knu.ac.kr (J.J.); yrlee@yu.ac.kr (Y.R.L.)
† These authors contributed equally to this work

Characterization methods

The calcium oleate, CCNPs, and CONPs were characterized by various physicochemical techniques such as X-ray diffraction (XRD), Fourier transforms infrared (FTIR) spectroscopy, scanning electron microscopy (SEM) with energy dispersive X-ray analysis (EDAX), and thermogravimetric analysis (TGA) with derivative thermogravimetry (DTG). The XRD patterns of CCNPs and CONPs were obtained on a Rigaku D/MAX-2500 diffractometer using Cu Kα as the radiation source (λ = 1.54 Å) and a liquid nitrogen-cooled germanium-based solid-state device as the detector. The XRD patterns of the CCNPs and CONPs were recorded in the scattering angle 2θ range of 5–80º, at a step interval of 0.02º and an integration time of 5s at each step. The FTIR spectra were recorded in transmittance mode on a Shimadzu IRPrestige-21 FTIR spectrometer in the wavenumber range of 400–4000 cm^{-1} by the co-addiction of 50 scans at a resolution of 16 cm^{-1}. The FTIR analyses were carried out at room temperature using potassium bromide (KBr) pellets containing around 1% of the solid nanoparticle samples, which were compacted in a uniaxial press under nominal pressure of 60 kN. The spectrum of dry KBr was recorded for background subtraction before recording the samples. The thermal stability of the samples was measured by means of TGA with a TA Instruments SDT Q600 setup. Each sample was heated from 25 to 1000 ºC at a scanning rate of 10 ºC/min under an air atmosphere. SEM images with EDAX were performed on a Hitachi S-4800 with an acceleration voltage of 4 kV by placing the CCNPs and CONPs on a conductive carbon tape.

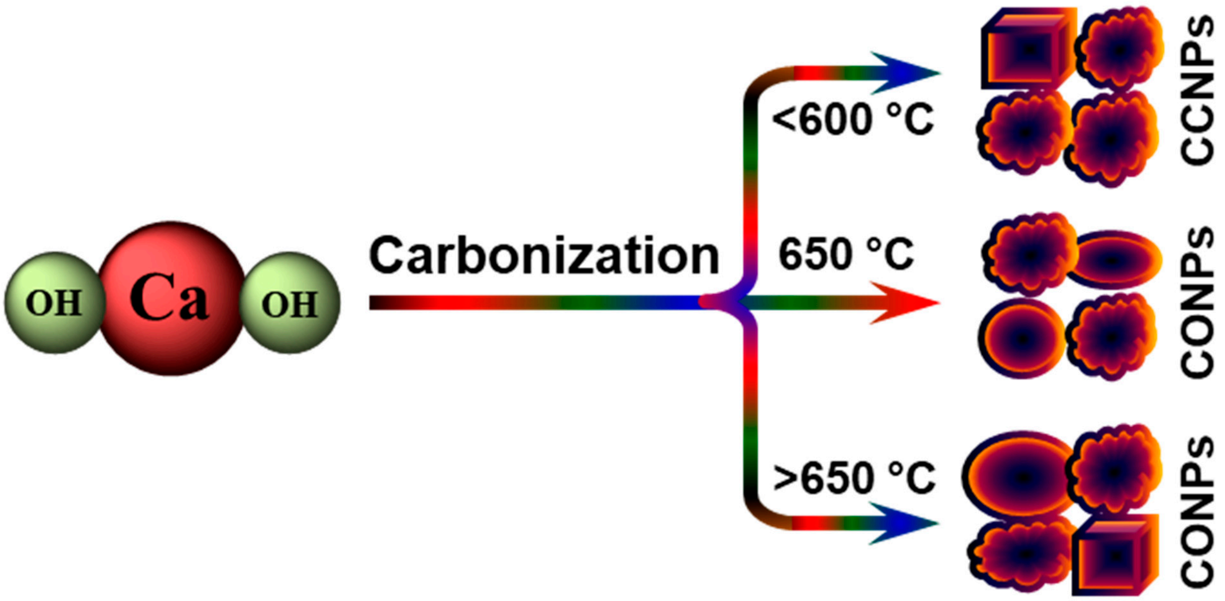

Scheme S1: Illustrate the formation of CCNPs and CONPs from calcium hydroxide via simple carbonization/calcination.

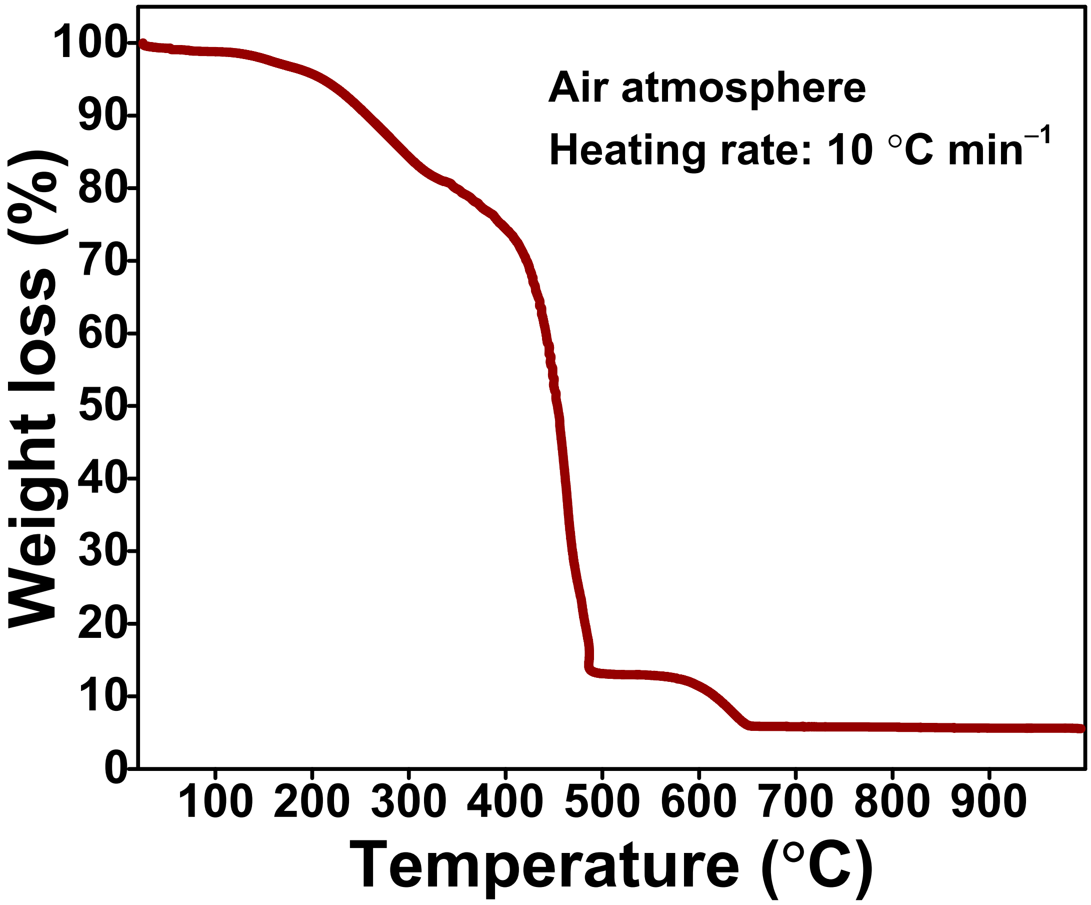

Figure S1: TGA curve of calcium oleate was synthesized using calcium hydroxide and oleic acid.

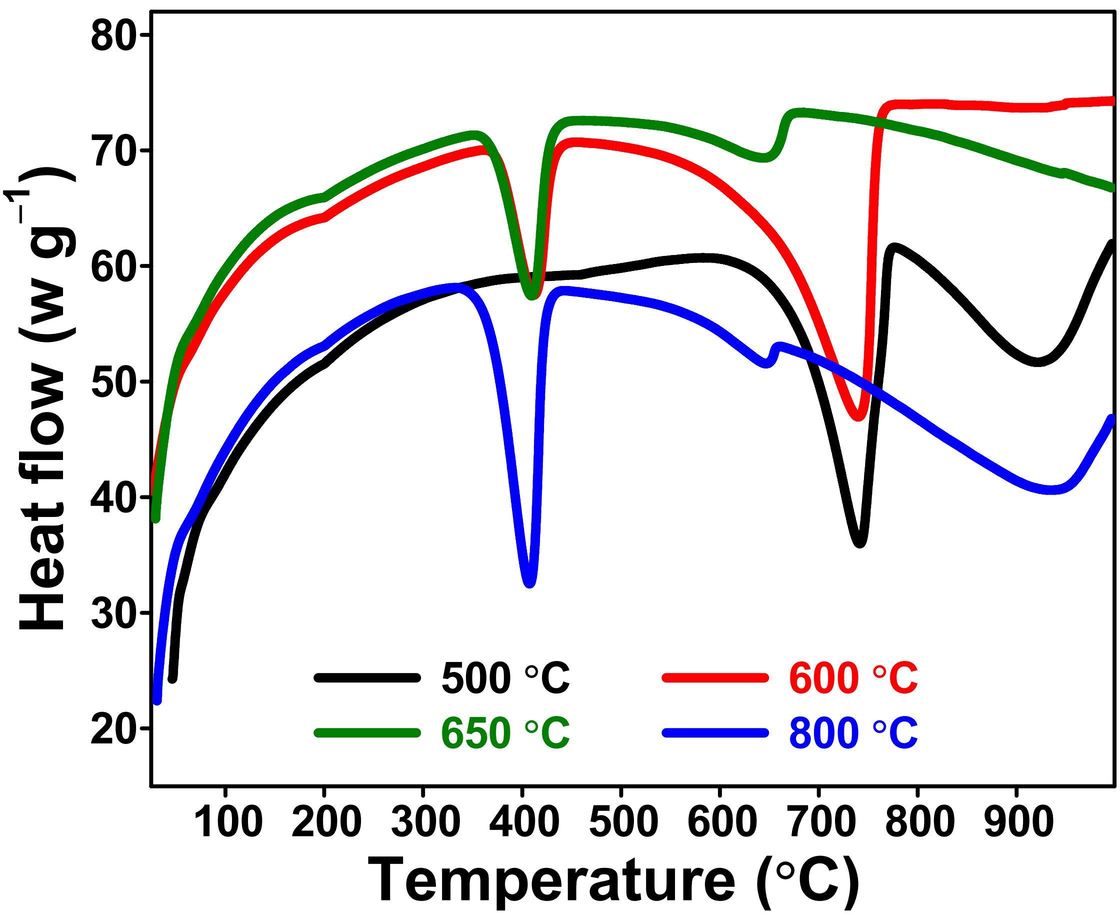

Figure S2: DTG curves of CCNPs and CONPs were obtained from calcium oleate.

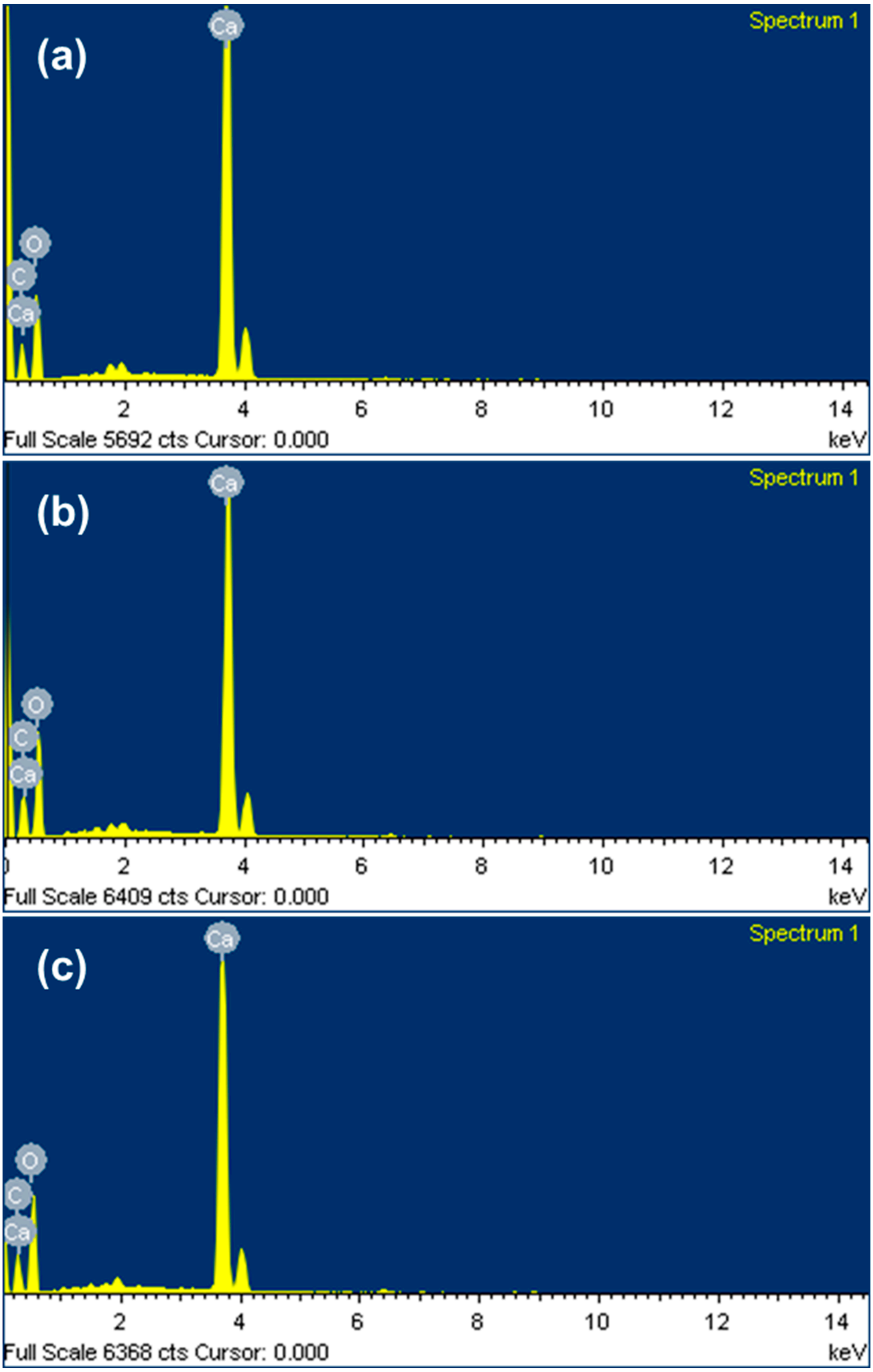

Figure S3: EDAX spectra of (a) CCNPs (550 °C), (b) CONPs (650 °C) and (c) CONPs (750 °C) synthesized using calcium oleate.

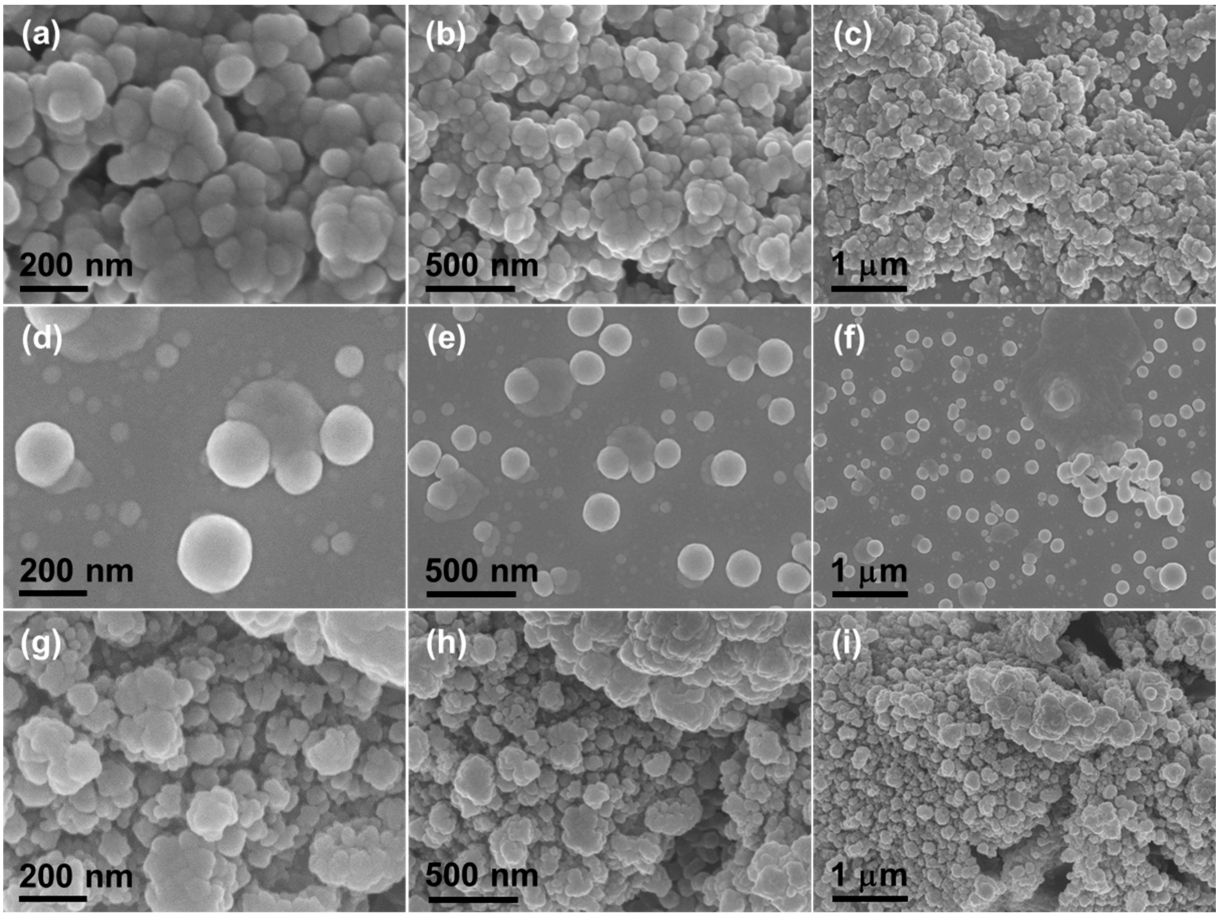

Figure S4: (a-c) SEM images of CCNPs were synthesized at 550 °C using calcium hydroxide. SEM images of CONPs synthesized at 650 °C (d-f), and 750 °C (g-i) using calcium hydroxide.
